# Do ultrasonic instrumentation and air polishing procedures adversely affect dental restorations?

**DOI:** 10.1038/s41432-025-01169-w

**Published:** 2025-06-10

**Authors:** Annabell Harman, Bryan Murchie

**Affiliations:** 1 General Professional Trainee, Newcastle Dental Hospital, Newcastle upon Tyne, UK; 2 Clinical Lecturer in Restorative Dentistry, School of Dental Sciences, Newcastle University, Newcastle upon Tyne, UK

**Keywords:** Dental caries, Dental biomaterials, Bonded restorations, Periodontitis

## Abstract

**A Commentary on:**

**Esati J, Amran T, Weiger R, Alsulaimani L, Blatz M B, Eggmann F**.

Adverse effects of ultrasonic instrumentation and air polishing on dental restorations: a systematic review of laboratory studies. *J Esthet Restor Dent* 2025; 10.1111/jerd.13428.

**Objective:**

A systematic review of the literature was conducted, assessing the potential for adverse effects on surface roughness and marginal integrity with use of ultrasonic instrumentation and air polishing on variety of dental restorations. With the aim to guide clinical practice to aid mitigation of adverse effects.

**Data sources:**

Five databases: Cochrane Library, OpenGrey through DANS, PubMed, Scopus, and Web of Science, and supplemental manual searches were used to identify relevant literature. The review adhered to the PRISMA guidelines.

**Study selection:**

Publications were included between 1978 and 2022. Population: dental restorations or restorative biomaterials in vitro. Intervention: ultrasonic instrumentation and/or air polishing. Comparison: no debridement procedure or paste polishing. Outcome: surface roughness and/or marginal quality. Forty-two laboratory studies were included in the final analysis. The studies evaluated data to answer the following research question: In specimens made of or featuring dental restorations or restorative biomaterials, how does the use of ultrasonic instrumentation and/or air polishing, compared with no debridement procedure or paste polishing, affect the surface roughness and/or marginal quality?

**Data extraction and synthesis:**

Data extracted included: author(s) and year, laboratory studies, biomaterials evaluated, specimen quantity, specimen geometry, grouping methodology, types of ultrasonic and/or air polishing devices used, air polishing powders, device operational settings, application type and duration of ultrasonic and/or air polishing, assessments of surface roughness and marginal quality, and the observed effects on both surface roughness and marginal quality. Biomaterial assessed included: porcelain fused to metal (PFM), zirconia (ZrO_2_), lithium disilicate (LDS), polymer-infiltrated ceramic network material (PICN), fine-structure felspathic ceramic (FSFC), gold alloy, amalgam, resin-modified glass ionomer cements (RMGIC), conventional and flowable resin-based composite (RBC), and silorane-based RBCs restorations. Studies measured surface roughness via contact profilometry, scanning electron microscopy, and atomic force microscopy. Ra was used as a parameter for surface roughness. Stereomicroscopy and confocal laser microscopy were used to assess marginal quality. Risk of bias was assessed using the RoBDEMAT tool.

**Results:**

Ultrasonic instrumentation and air polishing both negatively impacted surface roughness. Air polishing with sodium bicarbonate and calcium carbonate powders had a significantly greater effect on surface roughness, compared with erythritol and glycine powders. The surface roughness for RBCs and RMGICs were the most affected by ultrasonic instrumentation. In comparison, ZrO_2_ and LDS restorations were found to have the highest level of resistance compared to other restorations. Three studies observed that an increase in surface roughness could be mitigated using rubber cups and polishing paste after instrumentation. Ultrasonic instrumentation and air polishing methods both led to an adverse marginal quality outcome. Crowns with a narrow ceramic shoulder margin (0.7 mm) were particularly susceptible. RMGIC and amalgam restorations had a greater adverse impact on the marginal quality compared with RBC restorations.

**Conclusions:**

It is advised to use air polishing with less abrasive powders, including erythritol and glycine, to mitigate surface damage and changes at the marginal interface. Another important clinical consideration is the type of restorative material. High strength ceramic restorations, such as LDS and ZrO_2_, are much more resistant to surface roughening compared with all other materials.

## GRADE Rating:



## Commentary

Ultrasonic instrumentation and air polishing are both routine methods for periodontal disease treatment, used to remove biofilm, calculus, and staining^1^. Currently, reviews have compared ultrasonic instrumentation and air polishing efficiency for periodontal management^2^, and the dental literature has independently evaluated the outcomes of ultrasonic instrumentation, and air polishing, on dental restorations. However, no systematic reviews to-date have directly compared both methods regarding any potential adverse effects on direct and indirect restorations. Previously published laboratory studies have indicated that ultrasonic instrumentation and air polishing may detrimentally affect the marginal integrity and surface roughness of restorations. The clinical implications can lead to plaque accumulation, with an increased risk of periodontal inflammation and caries. This impacts the overall aesthetic outcome and restoration longevity^3^, including associated symptoms such as pain/sensitivity. This current systematic review^4^, therefore, aims to address a current gap in knowledge, analysing the effect of both ultrasonic and air polishing methods when used on direct and indirect restorations.

This review paper provided useful findings that could be translated across to use in clinical practice. It was found that ultrasonic instrumentation led to a significant increase in surface roughness, alongside marginal degradation. Roughness changes infer permanent damage has taken place on the restoration surface. Comparable, or even worse, findings were reported following air polishing with sodium bicarbonate and aluminium trihydroxide materials. But, by comparison, air polishing with erythritol and glycine led to a significantly reduced abrasive outcome, particularly for composite and RMGIC restorations. This suggests that the debridement process using less abrasive powders is the preferred mode of treatment to mitigate irreversible damage on the restoration surface present within the immediate vicinity of the periodontal treatment site.

The detrimental effects of ultrasonic and air polishing on direct restorations could also influence the clinical decision-making process regarding the selected restorative material. Under specific circumstances, indirect materials may be a sensible approach for periodontally susceptible patients who require regular periodontal reviews and treatment. However, this approach is highly case-dependent, as indirect restorations are considerably more invasive with removal of up to 76% of the tooth structure^5^. Especially as both PFM and feldspathic ceramic materials were found to require greater shoulder preparations (1.5 mm) to reduce susceptibility to irreversible marginal damage. Clinical judgment of periodontal health must be weighed up against other important risk factors, including pulp devitalisation. It would be the overall preference of the authors to be minimally invasive, where at all possible. Prioritising periodontal health, but with emphasis of an awareness of restorative materials to avoid unnecessary iatrogenic damage. Subsequently, post-operative surface roughness should be mitigated with a rubber cup and polishing paste, including minor additions as required. Importantly, this raises a key awareness of the consenting process for periodontal management, which should always include potential restoration adverse effects, in addition to costs for refurbishment/replacement.

Bias was measured thoroughly. The RoBDEMAT toolkit was used, which was developed by experts specifically for pre-clinical dental materials research to improve their quality and their systematic reviews^6^. Duplicate studies were removed, and two investigators independently screened the titles and abstracts of the reference list of study reports and used the RoBDEMAT toolkit for each individual study. Differences in opinion for inclusion eligibility were resolved by a third investigator. Investigators concluded that some degree of bias existed within the studies, particularly issues with randomization and control groups, sample size justification, and blinding.

Overall, this was a comprehensive and robust systematic review. A thorough search of five databases (Cochrane Library, Web of Science, PubMed, Scopus, and OpenGrey) was undertaken. However, one major limitation was the exclusive inclusion of *in vitro* studies, which analysed restorations under optimal conditions without other clinically relevant factors being considered. Although this was an intentional design, it was not made entirely clear why this approach was chosen. Clinically relevant studies would have been more suitable for direct translation to clinical practice. It is, therefore, recommended that the findings from this paper be interpreted with caution.

The timescale of all studies included ranged between 1978 and 2022, covering a large period of dentistry, during which time most biomaterials have significantly changed/advanced. The inclusion of earlier versions of biomaterials is unlikely to provide an accurate reflection of modern successors, indicating a degree of heterogeneity. It would have been useful to further clarify/investigate any differences in the material performance as a function of time. It could be assumed that modern materials are comparatively more resistant to change following ultrasonic and air polishing treatments, further complicating a clinical understanding and application to routine practice.

## Summary

This systematic review demonstrated that mechanical ultrasonic instrumentation and air polishing will cause significant adverse outcomes to the surface roughness, and marginal integrity, for both direct and indirect restorations. Only air polishing with erythritol and glycine powders mitigated damage to direct restoration surfaces. However, these findings should be interpreted with caution for clinical practice, as only in vitro studies were included for analysis. Furthermore, not all studies represented a relevance to modern restorative materials.

Practice points

- Air polishing with erythritol and glycine powders will help mitigate changes to the surface roughness and marginal integrity.
- High strength ceramics, such as zirconia and LDS, are most resistant to surface change.
- Composite and RMGIC, including thin ceramic margins, are most susceptible to changes from air polishing and ultrasonic treatments.
- Consideration should be given to post-operative treatment with rubber cups and polishing paste to help mitigate post-debridement roughness.
